# Evaluating EEG Utilisation and Diagnostic Yield in a Psychiatry-Led Service: An Audit From a Low- and Middle-Income Country

**DOI:** 10.1192/bjo.2026.11567

**Published:** 2026-06-30

**Authors:** Kaynat Riaz, Imtiaz Ahmad Dogar, Duaa Fatima, Yahya Anwaar

**Affiliations:** Allied II Hospital, Faisalabad Medical University, Faisalabad, Pakistan

## Abstract

**Aims:**

Electroencephalography (EEG) is frequently used within psychiatric services to support diagnostic clarification in presentations such as first-episode psychosis, suspected seizure disorders, dissociative episodes, catatonia, and complex neurodevelopmental conditions. In many low- and middle-income countries (LMICs), EEG services are embedded within psychiatry departments and support both psychiatric and neurological referrals. This audit aimed to evaluate EEG utilisation, documentation quality, and diagnostic yield within a psychiatry-led service, benchmarked against international standards, and to identify areas for quality improvement.

**Methods:**

A retrospective service audit was conducted of all EEGs performed between 2019 and 2024 in the Department of Psychiatry and Behavioural Sciences at Allied II Hospital, Faisalabad, Pakistan. The department provides EEG services for both psychiatric and neurological referrals. Data were extracted from the departmental EEG reporting system and analysed using Python and Microsoft Excel. Variables examined included annual EEG volumes, recording duration, wake versus sleep recordings, diagnostic outcomes, and completeness of clinical documentation. Findings were benchmarked against guidance from the International Federation of Clinical Neurophysiology (IFCN), American Clinical Neurophysiology Society (ACNS), and NICE.

**Results:**

A total of 2,670 valid EEGs were analysed over 6 years. Annual EEG volumes remained stable, including during the Covid-19 pandemic, demonstrating sustained service delivery, with activity increasing to 503 EEGs in 2024. Overall, 13.9% (n=405) of EEGs showed abnormal findings, 85.8% (n=2,494) were normal, and 0.2% had unclear outcomes, consistent with expected diagnostic yield. Wake EEGs accounted for 69.8% of recordings, while 28.4% included sleep recordings, indicating appropriate use for paediatric and complex cases; sleep-wake state was unclear in only 1.6% (n=47). The mean EEG duration was 25.2 minutes (median 19.0 minutes, IQR 16–23 minutes) showing satisfactory adherence to standards, with some recordings below recommended minimum durations. Documentation quality was generally strong, with missing data only limited to weight 12.8%, height 4.0% and gender 0.4%

**Conclusion:**

This audit demonstrates that EEG services delivered within a psychiatry-led department in an LMIC largely comply with international standards, with appropriate diagnostic yield and sustained utilisation over six years. Overall documentation quality was high, Identified areas for quality improvement include ensuring minimum recording durations, improving structured data entry, and refining referral practices to optimise diagnostic efficiency. An action plan has been implemented, with re-audit planned within 12–18 months to assess impact.

